# Preparation of Propanols by Glycerol Hydrogenolysis over Bifunctional Nickel-Containing Catalysts

**DOI:** 10.3390/molecules26061565

**Published:** 2021-03-12

**Authors:** Alexander A. Greish, Elena D. Finashina, Olga P. Tkachenko, Leonid M. Kustov

**Affiliations:** 1 N.D. Zelinsky Institute of Organic Chemistry, Leninsky Prospect, 47, 119991 Moscow, Russia; agreish@yandex.ru (A.A.G.); finesta@mail.ru (E.D.F.); ot@ioc.ac.ru (O.P.T.); 2 Chemistry Department, Lomonosov Moscow State University, Leninskie Gory, 1, bldg. 3, 119991 Moscow, Russia; 3 Laboratory of Nanochemistry and Ecology, Institute of Environmental Technologies & Engineering, National University of Science and Technology “MISiS”, Leninsky Prospect, 4, 119049 Moscow, Russia

**Keywords:** hydrogenolysis, glycerol, propanols, zirconia, titania, tungstena

## Abstract

The paper presents the results obtained in studying glycerol hydrogenolysis into 1-propanol and 2-propanol over bifunctional Ni/WO_3_-TiO_2_ and Ni/WO_3_-ZrO_2_ catalysts in the flow system. Due to the optimal combination of acidic and hydrogenation properties of the heterogeneous catalysts, they exhibit higher performance in glycerol conversion into C_3_ alcohols, although the process is carried out in rather mild conditions. At the reaction temperature of 250 °C and hydrogen pressure of 3 MPa, the total yield of 1-propanol and 2-propanol reaches 95%, and the glycerol conversion is close to 100%.

## 1. Introduction

Today an important direction for obtaining motor fuel from vegetable raw materials is the manufacturing of so-called “biodiesel”, which is a mixture of fatty acids esters. The production of this type of motor fuel includes, as the main stage, the transesterification of vegetable oils or animal fats using low molecular alcohols, with glycerol being formed as the main by-product [1,2,3]. In this regard, there is a problem of utilization of glycerol that is accumulated in large quantities [4,5,6,7,8,9,10].

One of the ways of processing glycerol can be catalytic hydrodehydroxylation of glycerol in the H_2_ atmosphere with the formation of such valuable products for the chemical industry as 1-Propanol (1-Pr) and 2-Propanol (2-Pr) that can be obtained by hydrogenation conversion of glycerol. However, the direct transformation of glycerol into simple alcohols is really a big challenge, with many research teams trying to solve this problem for the last decade [11,12,13,14]. The formation of C_3_ simple alcohols from glycerol under the hydrogenolysis conditions can be represented by the following chemical equation:

C_3_H_8_O_3_(l) + 2H_2_(g) = C_3_H_8_O(l) + 2H_2_O (1)

Table 1 shows the values for the standard enthalpy Δ*H^o^* and Gibbs free energy Δ*G^o^* for the glycerol conversion into 1-Pr and 2-Pr [15]. One can notice that the values of the Gibbs free energy for the formation of 1-Pr and 2-Pr by hydrogenolysis of glycerol are significantly negative, which means from the thermodynamics view that the reactions can proceed irreversibly.

Glycerol conversion into C_3_ simple alcohols under hydrogenolysis conditions is accompanied by the formation of two water molecules, as well as by the addition of two H_2_ molecules to the glycerol molecule.

Thus, the process should proceed through a series of consecutive steps, at least two dehydration steps and two hydrogenation steps. It is obvious that dehydration steps (or dehydroxylation steps) require the existence of strong Brønsted acid centers on the catalyst surface. 

At the same time, it is necessary to reduce unsaturated bonds that appear after removing two OH groups from a glycerol molecule by adding hydrogen, these hydrogenation steps are associated with the participation of metal centers activating hydrogen molecules. 

**Figure 1:**
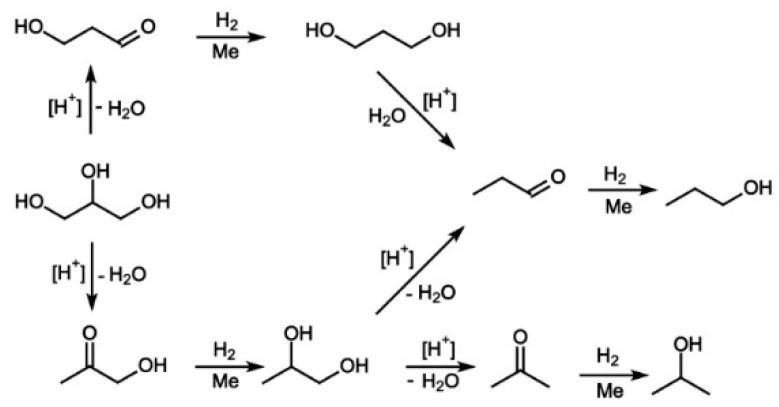
A possible mechanism of glycerol hydrogenolysis into 1-Pr and 2-Pr on a bifunctional catalyst.

A possible mechanism of glycerol conversion into 1-Pr and 2-Pr, which takes into account participation of two types of catalytic centers in the glycerol hydrogenolysis, is shown in Figure 1 [14].

In accordance with the reaction scheme, the catalyst should have bifunctional properties for the glycerol conversion into propanols, i.e., we are talking about the mechanism, which can be called the dehydration–hydrogenation mechanism [16,17]. 

Thus, the glycerol conversion into propanols is most likely carried out through consecutive reactions, where propanediols are formed as intermediate products. Obviously, a high selectivity for propanols can be achieved if the rates of dehydration and hydrogenation reactions proceeding on the catalyst surface exceed the rate of desorption of propanediols formed in the reaction as intermediates from the catalyst surface into the gas phase. This circumstance favors the decrease in the propanediols concentration in the vapor-gas flow passing through the catalyst bed and, consequently, in their amount in the reaction products.

Much attention is paid to developing an acidic carrier for the catalyst for glycerol hydrogenolysis. Some research groups pointed to the importance of acidic Brønsted centers in the occurrence of glycerol hydrogenolysis, in particular in 1,3-PD formation. It was demonstrated that the yield of 1,3-PD correlated with the concentration of the acidic Brønsted centers [11,18,19,20,21,22,23,24]. Very often the catalysts designed for glycerol hydrogenolysis contain tungsten oxides (WOx), which are capable of strengthening Brønsted acidity. In some works, the acidity promoted by tungsten oxides is considered to be a key factor in the 1,3-PD formation [18,25,26,27]. According to some research considering the glycerol hydrogenolysis mechanism, H-atoms obtained as a result of H_2_ dissociation are involved not only in the desorption of intermediate compounds, but also in the generation of the acidic Brønsted centers [23,28,29,30]. In 2010, Gong et al. [22] obtained Pt/WO_3_/TiO_2_ systems and found that the main role of WO_3_ was to regulate the Brønsted acidity of the catalyst.

In 2008, K Akurati et al. [31] supposed that during calcination of a binary WO_3_-TiO_2_ system, the formation of an oxygen bond between W^+6^ and Ti^+4^ cations as Ti^+4^-O-W^+6^ occurs. Due to the difference in the tungsten and titanium valences, the formation of such oxygen bond results in the appearance of anionic vacancies at the tungsten cations as well the simultaneous increase in the value of a positive charge on the titanium cations that cause the strengthening of the Lewis acidity. 

In 2013, Zhang et al. [23] developed a new method for the synthesis of Ti-W oxides and evaluated how the types of tungsten oxides affect the structure of the catalyst. According to the authors, the presence of strong Brønsted acidic centers is most likely responsible for the selectivity of glycerol hydrogenolysis to 1,3-PD. It should be noted that, in some cases of glycerol hydrogenolysis into propanediols carried out over catalysts on acidic carriers, 1-Pr is often detected, which is considered a byproduct, although sometimes the yield of this alcohol is relatively high. In particular, the formation of 1-Pr with a high yield was observed during glycerol hydrogenolysis carried out in the aqueous solution over a catalyst which consisted of Ru/C + Amberlyst [17].

In 2010, Qin et al. [25] reported that the yields of 1-Pr and 2-Pr were 56.2% and 5.3%, respectively, with the glycerol conversion of 84.5% during glycerol hydrogenolysis in the reactor with a fixed bed of the 4%Pt/WO_3_-ZrO_2_ catalyst (containing 10 wt.% W and calcined at 700 °C) at 130 °C and 4 MPa of H_2_. In this study, a 60% aqueous solution of glycerol was used. This process is considered to be expensive due to the use of platinum, while the yields of propanols are not high enough and are mainly considered as byproducts in the preparation of the target 1,3-PD.

In 2010 and 2011, Tomishige et al. [32,33] reported that 1-Pr was obtained with yields of 21% and 24%, respectively, on the Ir/SiO_2_ catalyst modified by Re and a small addition of sulfuric acid at 120 °C and 8 MPa of hydrogen. In 2011, Thibault et al. [34] obtained a 18% yield of 1-Pr by glycerol hydrogenolysis at 200 °C and at an H_2_ pressure of 3.45 MPa using a homogeneous Ru complex and methanesulfonic acid in the mixed water–sulfolan solvent.

In 2012, Zhu and others [18] received a 33% yield of 1-Pr in glycerol hydrogenolysis carried out over a Pt-HSiW/SiO_2_ catalyst containing a heteropoly acid at 200 °C and 5 MPa of hydrogen. It was found that increasing hydrogen pressure promotes propanediol hydrogenolysis with formation of propanols. When using nickel, instead of Pt, the yield of 1-Pr decreased to 4% [11].

In the work [35], Pd and Fe metals were used in a combination. The obtained Pd–Fe bimetallic nanoparticles provided the yield of mono-alcohols at the level of 80% at complete glycerol conversion in 24 h at 240 °C and hydrogen pressure of 5 atm, with the ethanol selectivity reaching 70%, while the selectivity for 1-Pr and 2-Pr was only 10%.

When using two-layer catalysts (zirconium phosphate was loaded into the upper layer, and the Ru supported catalyst was charged in the lower layer) in a reactor with a fixed catalyst bed with a continuous flow for glycerol conversion into 1-Pr, Wang et al. [22] suggested a possible reaction pathway including hydrogenolysis of glycerol. It was found that the two catalytic systems with sequential layers can transform glycerol into 1-Pr with a 100% conversion of glycerol by the way of dehydration and subsequent hydrogenation, in this study Zr phosphate transformed glycerol into acrolein, whereas the Ru/SiO_2_ catalyst converted acrolein into 1-Pr.

As far as glycerol hydrogenolysis uses hydrogen as a hydrogenation reagent, the catalyst should be able to activate hydrogen molecules. It is well known that noble metals are able to perform this function rather efficiently and therefore they are widely used in hydrogenation catalysts. One can notice that rather expensive catalysts based on Rh, Ru or Pt and severe conditions were used in all the sources mentioned above. As a rule, all these catalytic systems require a high hydrogen pressure, as well as a sufficiently high content of the noble metal (up to 4–5 wt. %).

Despite Ni not being a precious metal, it can also be applied for hydrogenation reactions; however, Ni-containing catalysts are rarely employed in glycerol conversion into propanols, because the yield of these alcohols remains relatively low even in the process occurring in severe conditions. In the work in [13], a catalytic system capable of performing hydrogenation conversion of glycerol into 1-Pr and consisting of layers of a zeolite and a catalyst based on metallic Ni was developed. The catalysts were loaded into the reactor with a fixed catalyst bed. The reaction was carried out at 220 °C, and an H_2_ pressure of 20 atm. At the 90% glycerol conversion, the selectivity for 1-Pr was about 60%. At the same time, the liquid-phase hydrogenation of glycerol (240 °C, H_2_ pressure of 61 atm) with the use of the 1%Ni/HSiW/Al_2_O_3_ catalyst provided the glycerol conversion of 39% and the selectivity for 1-Pr was around 55%.

The authors of [14] prepared the catalysts capable of converting glycerol into simple alcohols, which consisted of nickel nanoparticles supported on oxide carriers (Ni/SiO_2_ or Ni/Al_2_O_3_), with a content of Ni nanoparticles of 45–55 wt. %. The process of glycerol hydrogenation into mono-alcohols over these catalysts was carried out by supplying a 60% aqueous solution of glycerol and hydrogen to a flow reactor at a temperature of 230–320 °C, a hydrogen pressure of 40–75 atm and aliquid hourly space velocity (LHSV) of 3.0 h^−1^. The selectivity for the formation of 1-Pr on the Ni/Al_2_O_3_ catalyst at 275 °C and a H_2_ pressure of 60 atm was about 12%, and on the Ni/SiO_2_ catalyst it was close to 14%. The selectivity to 1-Pr on the Ni/Al_2_O_3_ catalyst at 320 °C and the pressure was 60 atm was 35.3%, while on the Ni/SiO_2_ catalyst it was 42.8%.

The significant disadvantages of the mentioned method for glycerol hydrogenation using Ni/Al_2_O_3_ and Ni/SiO_2_ catalysts are the relatively low selectivity to 1-Pr formation, while the nickel content in the catalyst is high; also, the severe conditions of the process were used to obtain acceptable results, namely the hydrogen pressure was higher than 60 atm, the temperature was in the range of 275–320 °C.

For the effective occurrence of these reactions, the appropriate catalyst is not only required, but also specific conditions that would reduce the occurrence of reverse and side reactions should be created, providing that the high selectivity to the resulting mono-alcohols is reached. In particular, the process parameters such as temperature, hydrogen pressure, glycerol feed rate, contact time and others can play an important role in increasing the selectivity of reactions. The reaction conditions should be chosen so as to reduce side reactions leading both to low-molecular products, and to the substances having a high molecular weight.

Earlier we carried out a series of studies [36,37,38] related to nitration of benzene on heterogeneous catalysts. The goal of those works was to develop a catalyst that possesses superacidic properties comparable to those of sulfuric acid. During testing of a number of catalysts in the nitration of benzene, it was found that there are some binary oxide systems, such as WO_3_-TiO_2_, WO_3_-ZrO_2_, MoO_3_-TiO_2_ that can demonstrate the enhanced acidity. In the glycerol hydrogenolysis, as in the nitration reaction, the acidic properties of the catalyst play a decisive role. It was decided that the superacidic systems developed by us earlier are most suitable for the glycerol hydrogenolysis. Thus, the binary oxide composition WO_3_-TiO_2_ was chosen as an acidic carrier for the catalyst destined for the glycerol hydrogenolysis. The question of the hydrogenating component of the catalyst was not so acute. As such, metallic nickel was chosen, which on the one hand has good hydrogenating properties, and on the other hand is significantly cheaper than any noble metal. After carrying out a series of experiments on the optimization of the appropriate conditions for the glycerol hydrogenolysis, it was found that the catalyst prepared demonstrates a fairly high efficiency in conversion of glycerol into propanols, with the yield of the latter of about 94%. The data obtained were published in the form of a short report [39]. In spite of that it was decided to expand our study by using another catalyst, Ni/WO_3_-ZrO_2_, to compare these samples in their activity in glycerol hydrogenolysis and to choose the best one.

The main purpose of this work was to develop an active catalyst for the hydrogenolysis of glycerol into propanols, as well as the search for conditions for this process to increase the selectivity of the propanols formed. Our strategy was based on the use of heterogeneous bifunctional catalytic systems. Coprecipitated mixtures of WO_3_ with ZrO_2_ or TiO_2_ with a molar ratio of WO_3_:MeO_2_ = 1:4.5–1:4, respectively, were used as carriers for the catalysts. Due to the composition, the catalysts possessed strong Brønsted acidity. Ni nanoparticles supported on the mixed oxide carrier in the amount of 16–20% wt. were used as a hydrogenating component. The process was carried out in a hydrogen atmosphere in the flow reactor with a fixed catalyst bed, glycerol was fed to the reactor as a 30–50% aqueous solution.

## 2. Results and Discussion

The oxide compositions consisting of mixtures of co-precipitated oxides WO_3_-TiO_2_ and WO_3_-ZrO_2_ were chosen as carriers, which proved to exhibit strong acidic properties in a variety of alkylation, isomerization and other reactions, for instance in the liquid-phase nitration reaction, where they demonstrated the rather strong Brønsted acidity comparable to that of sulfuric acid [36,37,38]. The data presented in these papers show quite convincingly that in these binary systems, the highest acidity is exhibited by samples containing tungstena (about 18–20% mol.). Metallic nickel was used as a hydrogenating component of the catalyst, which is important because nickel is significantly cheaper than any of the noble metals. However, unlike the latter, nickel is able to be oxidized in the presence of air, which leads to a decrease in the hydrogenating activity of the catalyst. Therefore, just before the run, the catalyst was reduced directly in the reactor in a hydrogen flow at 300–350 °C. 

Table 2 presents the data on the elemental composition of catalysts 20%Ni/18%WO_3_-ZrO_2_ and 16%Ni/20%WO_3_-TiO_2_ obtained by the energy dispersive X-ray spectroscopy (EDS). For comparison, the elemental composition of catalysts calculated on the basis of the amounts of nickel, tungsten, zirconium, and titanium compounds used in their preparation is given as well. As can be seen from Table 2, the EDS data practically coincide with the calculated values, the deviation does not exceed 10%, thus indicating a fairly homogeneous composition of the catalysts obtained.

**Figure 2:**
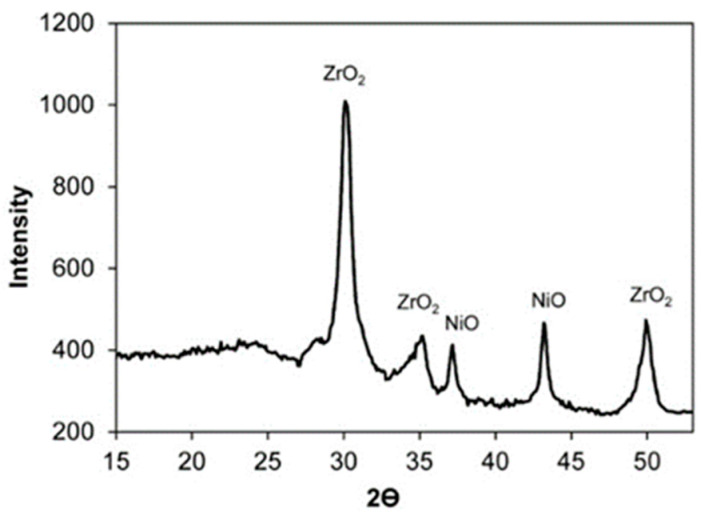
The XRD pattern of the 20% Ni/18%WO_3_-ZrO_2_ catalyst. The size of NiO nanoparticles is 25 nm, the size of ZrO_2_ nanoparticles is 13 nm.

**Figure 3:**
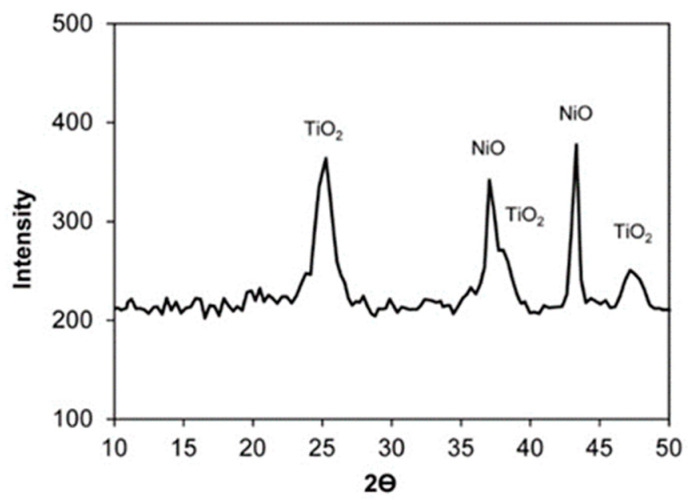
The XRD pattern of the 16%Ni/20%WO_3_–TiO_2_ catalyst. The size of NiO nanoparticles is 40 nm, the size of the TiO_2_ nanoparticles is 18 nm.

Figure 2 and Figure 3 present the X-ray patterns of the powders of the 20% Ni/18%WO_3_-ZrO_2_ and 16% Ni/20%WO_3_-TiO_2_ catalysts. 

There are reflexes corresponding to the ZrO_2_ and NiO phases in the XRD pattern of the 20%Ni/18%WO_3_-ZrO_2_ sample, while the XRD diffractogram of the 16%Ni/20%WO_3_-TiO_2_ catalyst exhibits the lines corresponding to the TiO_2_ and NiO phases. 

The absence of reflexes belonging to the WO_3_ phase in the XRD patterns of the studied catalysts can be explained by the rather low content of tungsten [40]. It can be assumed that, at such a concentration of tungsten, the crystalline phase of WO_3_ is not formed.

Most likely, in both catalysts, WO_3_ oxide is present in the form of an amorphous phase distributed in the phases of ZrO_2_ and TiO_2_ oxides, which are abundant in the catalyst composition. At the same time, the presence of WO_3_ does not practically affect the parameters of the crystal lattices of ZrO_2_ and TiO_2_ oxides.

The appearance of the NiO phase in the samples under consideration is quite expected, since nickel dispersed in a large amount on the catalyst surface is present in the oxidized form. To transfer nickel to the metal state, the catalyst was reduced in a hydrogen flow directly in the reactor before supplying the glycerol solution. Despite the high concentration of metal nickel, it is fairly evenly spread on the catalyst surface (see Supporting Information Figures S9 and S10)

To evaluate the acidic properties of the obtained catalysts, the method of diffuse reflectance infrared spectroscopy (DRIFT) was applied. In order to do this, the IR spectra of deuterated acetonitrile adsorbed on catalysts were measured, so as deuterated acetonitrile served as a probe molecule for the catalyst acidity [41]. Before measuring spectra, the samples were subjected to the thermal vacuum treatment at a temperature of 300 °C for 2 h to remove physically adsorbed water. Adsorption of deuterated acetonitrile was carried out at room temperature, at a saturated vapor pressure of CD_3_CN (96 Torr). 

The DRIFT method was used to study four samples of carriers: TiO_2_, 20%WO_3_-TiO_2_, ZrO_2_, and 18%WO_3_-ZrO_2_. Several absorption bands appear in the IR spectra in the course of adsorption of CD_3_CN on the studied samples, in particular, in the region of 2328–2292 cm^−1^, which is assigned to stretching vibrations of the C≡N bond in the acetonitrile molecule adsorbed on Lewis acid centers [42,43,44,45]. In addition, the absorption band at 2295–2258 cm^−1^ is present in the spectra, which corresponds to the coordination of acetonitrile molecules on Brønsted acid centers of the samples. The band at 2111 cm^−1^ presented in all spectra is attributed to the bending vibrations of C-D bonds in the CD_3_ group of acetonitrile.

**Figure 4:**
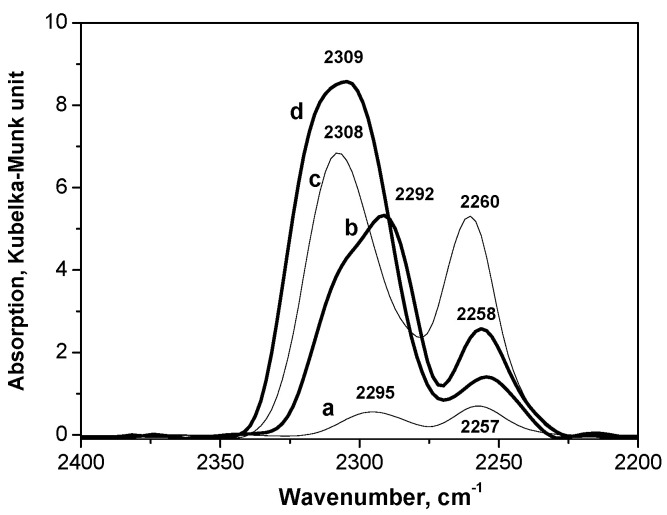
Diffuse reflectance infrared spectroscopy (DRIFT) spectra upon adsorption of CD_3_CN on ZrO_2_ (a), TiO_2_ (b), 18%WO_3_-ZrO_2_ (c), 20%WO_3_-TiO_2_ (d). Conditions: 96 Torr, 20 °C.

The value of the “blue shift” of the absorption band of stretching vibrations of the C≡N acetonitrile bond relative to the frequency of this bond in acetonitrile in the gas phase (2253 cm^−1^) can serve as an indicator of the acidity strength of Lewis centers [44]. Figure 4 shows a comparison of saturated vapor spectra of adsorbed acetonitrile on all studied carrier samples. Each spectrum is normalized to the maximum intensity of the absorption band in the region of 2300–2900 cm^−1^. According to the DRIFT data, the weakest Lewis acid centers exist on the TiO_2_ surface. The blue shift of the frequency of C≡N bonds stretching vibrations upon adsorption of acetonitrile in this case is 39 cm^−1^ (2292–2253 cm^−1^). Based on the DRIFT data, Lewis acid sites in the studied samples can be ranked according to their strength as follows: 20%WO_3_-TiO_2_ > 18%WO_3_-ZrO_2_ > ZrO_2_ > TiO_2_. Noteworthy is that the concentration of Lewis acid sites in zirconia is very low compared to other samples of the carriers. 

To evaluate qualitatively the binding energy of CD_3_CN with the carrier, IR spectra during the stepwise desorption of CD_3_CN from the samples at increasing temperature from 20 to 300 °C were obtained (see Supporting Information Figures S1–S4). The DRIFT spectra show that among the studied samples, the 20%WO_3_-TiO_2_ carrier exhibits the strongest adsorption of CD_3_CN at a higher temperature. When heating this sample in a vacuum at 300 °C for 30 min, a relatively large quantity of adsorbed D-acetonitrile remains on the carrier surface. According to the binding energy of CD_3_CN on the Lewis acid centers at an increased temperature that is favorable for CD_3_CN desorption, the samples can be arranged in the following sequence: WO_3_-TiO_2_ > WO_3_-ZrO_2_ ≈ TiO_2_ > ZrO_2_.

The spectroscopic data show that the addition of tungsten oxide to the carrier increases the strength of the Lewis acid sites. It is obvious that in the conditions of glycerol hydrogenolysis, when the catalyst contacts directly with water vapor, Lewis acid centers can be easily transformed into Brønsted acid sites, which can be presented by protons of water molecules adsorbed on transition metal cations or by protons of surface OH groups produced by dissociative adsorption of water on Lewis acid sites.

Additional information about the acidic properties of the samples, in particular, the presence of Brønsted acid sites, can be obtained from the analysis of changes in the IR spectra in the field of stretching vibrations of OH-groups (4000–3200 cm^−1^) associated with CD_3_CN adsorption (see Supporting Information Figures S5–S8). It is sufficient to compare the IR spectrum of the sample obtained during adsorption of CD_3_CN saturated vapor with the IR spectrum of the same sample heated in a vacuum (300 °C, 2 h) for complete removal of acetonitrile from its surface. Comparison of these spectra shows that the adsorption of acetonitrile on almost all samples is accompanied by the formation of hydrogen bonds of acetonitrile with OH groups located on the surface of the oxide carrier, which is a direct proof of the presence of Brønsted acid centers. The strength of Brønsted acid sites can be estimated by the low-frequency shift of the frequency of the OH groups of the sample after adsorption of acetonitrile (a wide absorption band with a maximum in the range of 3450–3200 cm^−1^). The frequency shifts for the stretching vibrations of surface OH groups for the studied oxide materials and SiO_2_ taken for comparison are presented in Table 3.

Based on these data, we come to the conclusion that double oxides such as 18%WO_3_-ZrO_2_ and 20%WO_3_-TiO_2_ exhibit much stronger Brønsted acidity compared to the pristine unmodified oxides. Thus, the addition of WO_3_ in the quantity of 18–20% mol. increases the Brønsted acidity strength of individual TiO_2_ and ZrO_2_ oxide carriers.

As noted above, a decisive role in the conversion of glycerol into propanols is assigned to the bifunctional nature of the heterogeneous catalysts used. Indeed, on the one hand, the catalyst should be responsible for glycerol dehydration proceeding with the participation of Brønsted acid centers of the catalyst, and, on the other hand, the catalyst should exhibit a hydrogenating function due to the presence of metal centers that can either activate molecular hydrogen or generate surface π-complexes with double bonds during hydrogenation reactions.

At the same time, the yield and selectivity of propanols are influenced not only by the composition and properties of the catalyst, but also by the conditions of the process, in particular, such parameters as the concentration of glycerol in the initial solution, the reaction temperature, pressure and the volume rate of hydrogen supply and the contact time. 

The main feature of this process is its occurrence in a two-phase system, namely with participation of liquid glycerol and gaseous hydrogen. As far as the temperature in the reaction zone is lower than the boiling point of glycerol, the latter passes through the catalyst bed as a liquid, and water of the solution evaporates and passes to the gas phase. In the process studied, hydrogen has two functions. On the one hand, hydrogen is a reagent that participates directly in the glycerol hydrogenolysis. On the other hand, hydrogen is a carrier gas, that promotes glycerol passing through the catalyst bed consisting of fairly small grains. It must be noted that at the reaction conditions hydrogen is fed with a ten-fold excess. A large amount of hydrogen is necessary to quickly remove propanols formed in the reaction from the catalyst bed; it is more favored by the fact that the boiling points of propanols are essentially lower than that of glycerol. As a result, fast desorption of propanols from the catalyst surface to the gas phase occurs. 

Since the glycerol viscosity is rather high, it is unlikely that glycerol penetrates inside micropores in the internal volume of the catalyst grains. Most probably, the reaction occurs due to the interaction of glycerol molecules with the active centers located at the outer surface of the catalyst. This suggests that the reaction rate should depend on the value of the outer surface of the catalyst, which, in turn, is determined by the size of the catalyst grains. We found that an increase in the size of the catalyst grains of more than 0.5 mm leads to a certain decrease in the conversion of glycerol. It is quite obvious that, at a relatively large grain size, the reaction rate may be limited by the rate of diffusion of glycerol into the internal volume of the catalyst. Therefore, in our runs, we used catalysts with fairly small grains, of 0.25–0.5 mm. 

**Figure 5:**
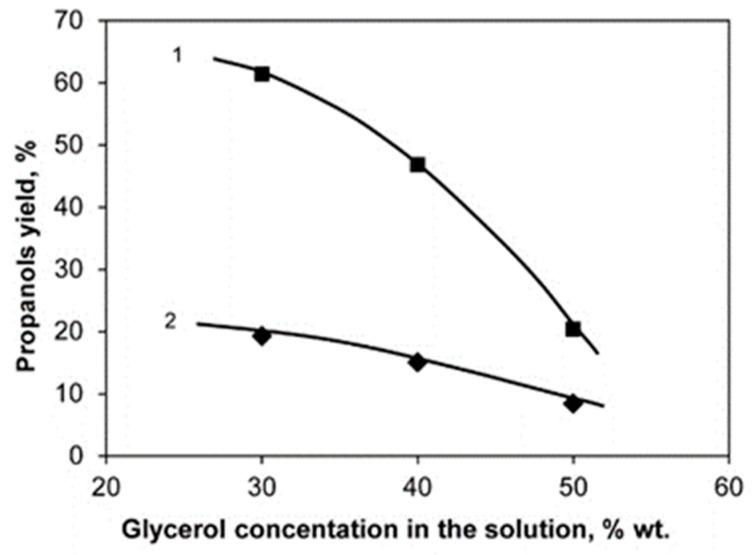
The dependence of the yield of 1-Pr (1) and 2-Pr (2) on the glycerol concentration in an aqueous solution at 100% glycerol conversion. Conditions: 20%Ni/18%WO_3_-ZrO_2_, Vcat. = 2 mL, T = 260 °C, LHSV = 1.66 h^−1^, P_H2_ = 2 MPa.

**Figure 6:**
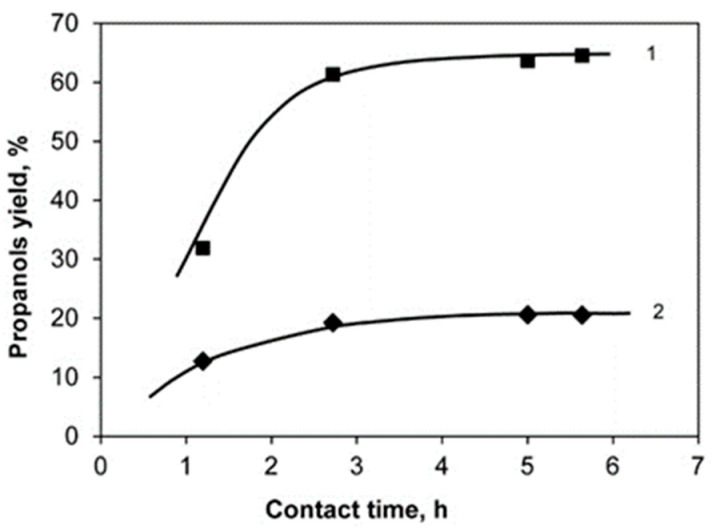
The dependence of the yield of 1-Pr (1) and 2-Pr (2) on the contact time. Conditions: the catalyst 20%Ni/18%WO_3_-ZrO_2_, 30% glycerol solution, T = 260 °C, P_H2_ = 2.55 MPa.

**Figure 7:**
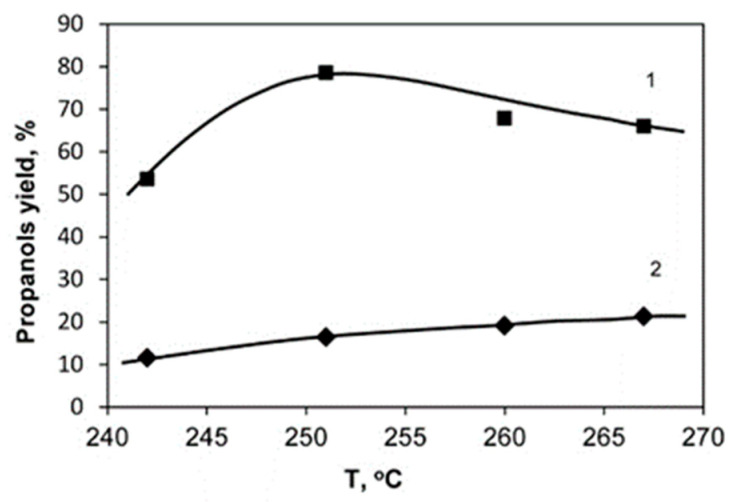
The dependence of the yield of 1-Pr (1) and 2-Pr (2) on the reaction temperature. Conditions: the catalyst 20%Ni/18%WO_3_-ZrO_2_, Vcat. = 2 mL, 30% glycerol solution, P_H2_ = 3.1 MPa.

Figure 5, Figure 6 and Figure 7 show the dependences of the yields of 1-Pr and 2-Pr on the concentration of glycerol in the initial solution, contact time and reaction temperature when the reaction is carried out over the 20%Ni/18%WO_3_-ZrO_2_ catalyst. 

The dependence of the target products’ yield on the glycerol concentration in the initial solution is of particular interest.

Experiments show that an increase in the concentration of glycerol in the initial solution leads to an increase in the yield of by-products, including heavy polyalcohols. Taking into account everything said above, it becomes obvious that the conditions for glycerol hydrogenolysis should be chosen to be very favorable for increasing the yield of propanols, and at the same time to minimize the yield of any by-products, including propanediols. From this point of view, it was most expedient to carry out the reaction in the conditions that ensure that glycerol is in a liquid state.

Indeed, in this case, due to the high viscosity, glycerol does not penetrate into the pores of the catalyst, and the reaction takes place exclusively on its outer surface. Then the main factor affecting the reaction rate is the value of the external catalyst surface, which directly depends on the size of the catalyst granules. In our experiments, catalysts with grain sizes of 0.25–0.50 mm were used.

Table 4 presents the results of the glycerol hydrogenolysis tests carried out over the 20%Ni/18%WO_3_-ZrO_2_ and 16%Ni/20%WO_3_-TiO_2_ catalysts. One can notice that there are certain conditions that allow us to obtain very high yields of propanols with the conversion of glycerol close to 100%. For example, for the 16%Ni/20%WO_3_-TiO_2_ catalyst, the maximum yield of propanols reaches 94.1%. 

According to experimental data obtained, the specified reaction conditions have a complex effect on the reaction proceeding. In particular, alcohols formed from glycerol have a lower boiling point than that of glycerol, as a result of which they can be removed from the reaction zone by a hydrogen flow. On the other hand, an increase in the hydrogen pressure in the reaction zone not only increases the rate of hydrogenation of double bonds, but at the same time reduces the rate of evaporation of intermediate propanediols, thereby increasing the likelihood of further conversion of propanediols into propanols. Additionally, the reaction temperature has a great influence on the yield of propanols. It can be seen from the data that there is an optimal temperature at which the yield of propanol-1 reaches the maximum value, which is equal to 250 °C. Thus, the results show that the activity of the catalysts is quite close, while the optimal conditions for the runs carried out over different catalysts are practically the same.

Under the optimum conditions, the main products are 1-Pr and 2-Pr, and the higher the conversion of glycerol simple alcohols, the higher the value of the 1-Pr/2-Pr ratio. For two catalysts studied, these values differ a little. For the catalyst 20%Ni/18%WO_3_-ZrO_2_, this ratio is in the range of 3–6, whereas for the catalyst 16%Ni/20%WO_3_-TiO_2_ it ranges within 9–14. Thus, the catalyst 16%Ni/20%WO_3_-TiO_2_ is characterized by a higher selectivity to 1-Pr formation. Noteworthy is that the carriers without nickel demonstrate a very low glycerol hydrogenolysis activity.

According to the reaction scheme given above, the transformation of glycerol into propanols passes two times through dehydroxylation. It would seem that the reaction should not finish at the propanols formation, and one could expect the third hydroxyl group of the glycerol molecule to be dehydroxylated also during the reaction, then the final product of the glycerol hydrogenolysis should be propylene. However, our attempts to detect by GLC some quantity of propylene in the gas flow coming out from the reactor were unsuccessful. So, the absence of propylene in the reaction products confirms that dehydroxylation of propanols formed in the reaction does not occur, most likely, because the hydrogen stream causes these alcohols to leave the catalyst without any further conversion.

There are a lot of papers related to the use of the binary oxide systems WO_3_–TiO_2_ and WO_3_–ZrO_2_ in various catalytic applications [46,47,48,49,50,51,52,53,54,55,56,57,58,59]. In most works, much attention is paid to the mechanism of the appearance of strong acidity in these oxide systems. It should be noted that these systems are able to exhibit strong acidity only in the case when they are prepared through wet-chemical techniques, in particular by means of sol–gel co-precipitation of the corresponding tungsten and titanium (or zirconium) hydroxides followed by calcination at a temperature of at least 500 °C [48]. Such essential increase in acidic properties of the double oxides does not occur when they are prepared by mixing of tungsten and titanium (or zirconium) oxides as powders. The authors [46] consider that coupling TiO_2_ with W^6+^ species should lead to an additional electronic state in the band-gap of nanocomposite particles, which in turn affects a change in the electronic properties and functionality of TiO_2_ itself. The main reasons were attributed to the mismatch of the TiO_2_ and WO_3_ band energies and formation of activating surface species such as a W^5+^ state, which acted as mediators for charge carrier separation. In the authors of [47]’s opinion, tungsten atoms enter as W^6+^ or W^4+^ cations into the rutile-type TiO_2_ lattice, by substituting the Ti^4+^ cations. While W^6+^-Ti^4+^ substitution leaves the surrounding rutile matrix unchanged, the W^4+^-Ti^4+^ substitution induces a local rutile-to-anatase transition. It was noticed [49] that the increasing surface acidity of WO_3_/TiO_2_ facilitates the adsorption of molecules containing hydroxyl groups, which obviously facilitates the enhancement of catalytic activity in the glycerol conversion. At the same time, it is emphasized that the activity of WO_3_/TiO_2_ strongly depends on such factors as the synthesis method and the amount of the tungsten dopant. 

The data on W surface-bulk distribution in WO_3_/ZrO_2_ samples prepared by various methods confirm the formation of a solid solution of W^6+^ ions in the zirconia lattice [52]. The difference of the WO_3_/ZrO_2_ lattice cell parameters calculated from the XRD data compared to pure ZrO_2_ also verifies the formation of the solid solution. Isolated WO_4_^2−^ tetrahedra attached to surface Zr^4+^ ions could form strong acidic sites according to the data on the surface W content. According to the data [53], as the electronegativity of the carrier cation increases (Al *>* Nb ∼ Ti *>* Zr), the electron density of the bridging W–O-support bond decreases and results in stronger acidic sites. The authors [56] consider the strong Brönsted acidity of WO_3_/ZrO_2_, which is explained by the formation of large zirconium polytungstate species on the WO_3_/ZrO_2_ surface. The superacidity exhibited by WO_3_/ZrO_2_ after heat treatment at 970–1020 K (H_0_ ≤ −14.52) is caused by the formation of strong Lewis acid sites containing pentacoordinated W^6+^ ions as a result of the dehydration of Brönsted acid sites. Complementary theoretical calculations of the Brønsted acidity of these Zr-WOx clusters have confirmed that they possess the lowest deprotonation energy values [57]. This insight provides a foundation for the future characterization and theory of acidic supported metal oxide catalytic materials that will, hopefully, lead to the design of more active and selective catalysts. 

## 3. Materials and Methods

### 3.1. Catalysts Preparation 

The WO_3_(18%)-ZrO_2_ carrier was prepared by precipitation with an ammonia solution of zirconium and tungsten hydroxides from an aqueous solution containing ammonium tungstate (NH_4_)_10_[H_2_W_12_O_41_] and zirconium oxychloride ZrOCl_2_. The resulting precipitate was washed with distilled water to a neutral reaction, dried at 150 °C for 2 h in air, and then calcined in an air flow at 520 °C for 3 h. In the resulting sample, the molar ratio of oxides WO_3_:ZrO_2_ was equal to 1:4.5.

The 20%Ni/[WO_3_(18%)-ZrO_2_] catalyst was prepared by impregnating the resulting carrier WO_3_-ZrO_2_ with an aqueous solution of nickel nitrate Ni(NO_3_)_2_•6H_2_O, followed by drying the catalyst at 120 °C for 2 h and calcination in an air flow at 350 °C for 2 h. Before the catalytic experiment, the catalyst loaded into the reactor was reduced in a hydrogen flow at 350 °C for 3.5 h. The content of metallic Ni in the catalyst was 20% mass.

The WO_3_(20%)-TiO_2_ carrier was prepared by precipitation with an ammonia solution of titanium and tungsten hydroxides from an aqueous solution containing ammonium dodecatungstate (NH_4_)_10_[H_2_W_12_O_41_] and titanium tetrachloride TiCl_4_. The resulting precipitate was washed with distilled water until it reached a neutral reaction, dried in air at 150 °C for 2 h and calcined in an air flow at 520 °C for 3 h. The molar ratio of oxides WO_3_:TiO_2_ in the resulting sample was 1:4.

The 16%Ni/[WO_3_(20%)-TiO_2_] catalyst was prepared by impregnating the WO_3_-TiO_2_ carrier with an aqueous solution of nickel nitrate Ni(NO_3_)_2_•6H_2_O, followed by drying the catalyst at 120 °C for 2 h and calcination at 350 °C for 2 h. Before the catalytic tests, the catalyst loaded into the reactor was reduced in a hydrogen flow at 350 °C for 3.5 h. The content of metallic Ni in the catalyst was 16% mass.

### 3.2. Catalysts Characterization

Diffuse reflectance IR Fourier-transform spectra (DRIFT) were recorded at room temperature using a NICOLET “Protege” 460 spectrometer equipped with a diffuse reflectance attachment [28] in the range 6000–400 cm^−1^ with a step of 4 cm^−1^. To reduce the signal-to-noise ratio, accumulation of 500 spectra was performed. CaF_2_ powder was used as a standard. Five samples were studied: TiO_2_, WO_3_(20%)-TiO_2_, ZrO_2_, WO_3_(18%)-ZrO_2_ and the catalyst 16%Ni/20%WO_3_-TiO_2_ (after the reaction).

Before measuring spectra, the samples were subjected to thermal vacuum treatment at a temperature of 300 °C for 2 h (the heating rate was 5°/min) to remove physically adsorbed water. Deuterated acetonitrile was used as a test molecule for acid sites. Adsorption was carried out at room temperature and saturated vapor pressure of CD_3_CN (96 Torr).

The elemental composition of the catalysts was determined using a scanning electron microscope SNE-3200M manufactured by SEC, combined with a system of the energy dispersive X-ray microanalysis QUANTAX manufactured by Bruker.

The X-ray phase analysis of the samples was performed using a DRON-2 diffractometer.

### 3.3. Catalyst Testing 

Experiments on the hydrogenolysis of glycerol were carried out in a flow reactor. The catalytic reactor was a stainless-steel tube with an inner diameter of 7 mm. The catalyst portion (2 g) with a particle size of 0.25–0.5 mm was loaded into the central part of the reactor. The free volume of the reactor was filled with ground quartz with a particle size of 0.5–1.0 mm. An aqueous solution of glycerol with a concentration of 30–50% was fed to the reactor by means of a syringe pump, the LHSV of the glycerol solution varied in the range of 0.46–1.66 h^−1^. The contact time (τ) was calculated by the formula: τ = v_cat_/LHSV, where v_cat_ is the catalyst volume. The process was carried out in the temperature range of 240–270 °C. The temperature in the reactor was kept constant using an electronic controller. The hydrogen pressure in the reactor was varied in the range of 2.0–3.6 MPa. The feed rate of hydrogen in all experiments was 40 cm^3^/min. Effluent products entered an ice-cooled separator in which separation of the liquid phase from the gas proceeded efficiently. Liquid samples for analysis were taken every 20–30 min. Analysis of the reaction products was carried out by the Gas chromatography (GC) method with a chromatograph Chromatek-Crystal-5000 equipped with a flame ionization detector using a capillary column with a free fatty acid phase (FFAP). (See Supporting Information Figure S11).

## 4. Conclusions

The present work demonstrates that the catalysts 20%Ni/18%WO_3_-ZrO_2_ and 16%Ni/20%WO_3_-80%TiO_2_ used in glycerol hydrogenolysis provide a propanols yield reaching 93–94%. Such an extraordinary result can be explained by the optimal ratio of the two functions in the catalysts prepared on co-precipitated oxides WO_3_-ZrO_2_, WO_3_-TiO_2_, which possess rather strong Brønsted acidity, providing high dehydrating ability. In turn, the presence of a large amount of Ni nanoparticles supported on the catalyst surface creates the conditions that are very favorable for hydrogenation stages proceeding with a high efficiency. It is clear that in order to improve the selectivity for propanols, it is necessary to optimize the reaction conditions so as to reduce the yield of by-products formed during the processes due to both destructive hydrogenolysis and condensation of initial glycerol. In turn, the use of an excessive amount of hydrogen reduces the likelihood of the dehydroxylation of propanols themselves with forming of propylene, due to the rapid removal of these alcohols by the hydrogen flow from the reaction zone. 

## Figures and Tables

**Table 1:** The values of the standard enthalpy (Δ*H^o^*) and Gibbs free energy (Δ*G^o^*) for 1-Propanol (1-Pr) and 2-Propanol (2-Pr) formation during glycerol hydrogenolysis.

Alcohol	Phase	Δ*H^o^*, kJ/mol	Δ*G^o^*, kJ/mol
1-Propanol	liquid	−206	−166
2-Propanol	liquid	−221	−178

**Table 2:** The composition of the 20%Ni/18%WO_3_-ZrO_2_, 16%Ni/20%WO_3_-TiO_2_ catalysts.

Catalyst	Element	Calculated Values		EDS Data	
		% mass.	% at.	% mass.	% at.
Ni/WO_3_-ZrO_2_	O	23.7	63.4	21.2	59.7
	Ni	19.0	13.8	20.7	15.8
	Zr	39.7	18.7	41.3	20.4
	W	17.6	4.1	16.8	4.1
Ni/WO_3_-TiO_2_	O	29.8	65.3	28.4	64.2
	Ni	15.3	9.1	17.2	10.5
	Ti	28.0	20.5	26.0	19.7
	W	26.9	5.1	28.4	5.6

**Table 3:** Frequency shifts of stretching vibrations of surface OH groups caused by deuteroacetonitrile adsorption.

Sample	Frequency of OH Groups Hydrogen-Bonded with Acetonitrile, cm^−1^	Frequency Shift of OH Groups Hydrogen-Bonded with Acetonitrile, cm^−1^
TiO_2_	3400–3450	~220–270
ZrO_2_	Very low intensity	-
WO_3_-TiO_2_	3200	~500
WO_3_-ZrO_2_	3150	~500
SiO_2_	3750	~375

**Table 4:** The results of the glycerol hydrogenolysis over the 20%Ni/18%WO_3_-72%ZrO_2_ and 16%Ni/20%WO_3_-80%TiO_2_ catalysts.

Catalyst	T, °C	P_H2_, MPa	LHSV, h^−1^	Glycerol conversion, %	Yield of 1-Pr, %	Yield of 2-Pr, %	ΣPropanols Yield, %
	251	3.60	0.69	100	61.4	13.1	74.5
Ni/WO_3_-ZrO_2_	261	3.00	1.38	99.9	58.8	16.4	75.2
	260	2.55	0.83	100	61.3	19.2	80.6
	260	3.18	0.48	100	63.6	19.1	82.7
	260	2.60	0.55	100	63.7	20.6	84.3
	267	3.15	0.55	99.9	66.0	21.2	87.2
	251	3.10	0.50	99.9	80.2	14.6	93.8
Ni/WO_3_-TiO_2_	255	3.05	0.69	100	76.2	8.5	84.6
	251	3.06	0.55	100	82.0	6.9	88.8
	246	3.07	0.47	100	82.2	6.9	89.1
	246	3.07	0.69	100	81.6	6.7	88.3
	246	3.07	0.47	100	85.8	6.3	92.1
	248	3.13	0.46	100	87.2	6.4	93.5
	251	3.15	0.47	100	87.8	6.4	94.1

Conditions: the flow reactor, 30% Glycerol solution, Vcat. = 2 mL, LHSV = 0.46–1.38 h^−1^, U_H2_ = 15 mL/min.

## Data Availability

Not applicable.

## Supplementary Materials

The following are available online: data on DRIFT spectra of CD_3_CN desorption from the ZrO_2_, TiO_2_, 18%WO_3_-ZrO_2_, 20%WO_3_-TiO_2_ samples at increased temperature, Figure S1: DRIFT spectra of CD_3_CN adsorption over ZrO_2_. 1—CD_3_CN, 20 °C, 96 Torr; 2—vacuum, 20 °C, 1 h; 3—vacuum, 100 °C, 30 min, Figure S2: DRIFT spectra of CD_3_CN adsorption over 18%WO_3_-ZrO_2_. 1—CD_3_CN, 20 °C, 96 Torr; 2—vacuum, 20 °C, 1 h; 3—vacuum, 100 °C, 30 min; 4—vacuum, 200 °C, 30 min; 5—vacuum, 300 °C, 30 min, Figure S3: DRIFT spectra of CD_3_CN adsorption over TiO_2_. 1—CD_3_CN, 20 °C, 96 Torr; 2—vacuum, 20 °C, 1 h; 3—vacuum, 100 °C, 30 min; 4—vacuum, 200 °C, 30 min; 5—vacuum, 300 °C, 30 min, Figure S4: DRIFT spectra of CD_3_CN adsorption over 20%WO_3_-TiO_2_. 1—CD_3_CN, 20 °C, 96 Torr; 2—vacuum, 20 °C, 1 h; 3—vacuum, 100 °C, 30 min; 4—vacuum, 200 °C, 30 min; 5—vacuum, 300 °C, 30 min, data on DRIFT spectra of the ZrO_2_, TiO_2_, 18%WO_3_-ZrO_2_, 20%WO_3_-TiO_2_ samples in the field of stretching vibrations of OH-groups (4000–3200 cm^−1^) obtained before and after CD_3_CN adsorption, Figure S5: DRIFT spectra of the TiO_2_ sample obtained before and after adsorption of CD_3_CN: 1—vacuum, 300 °C, 2 h; 2—CD_3_CN, 20 °C, 96 Torr, Figure S6: DRIFT spectra of the ZrO_2_ sample obtained before and after adsorption of CD_3_CN: 1—vacuum, 300 °C, 2 h; 2—CD_3_CN, 20 °C, 96 Torr, Figure S7: DRIFT spectra of the 20%WO_3_-TiO_2_ sample obtained before and after adsorption of CD_3_CN: 1—vacuum, 300 °C, 2 h; 2—CD_3_CN, 20 °C, 96 Torr, Figure S8: DRIFT spectra of the 18%WO_3_-ZrO_2_ sample obtained before and after adsorption of CD_3_CN: 1—vacuum, 300 °C, 2 h; 2—CD_3_CN, 20 °C, 96 Torr. SEM images of the outer surface of the Ni/WO_3_-TiO_2_ and Ni/WO_3_-ZrO_2_ catalysts obtained by the scanning electron microscope SNE-3200M (SEC), Figure S9: SEM image of the 16%Ni/(20%WO_3_-TiO_2_) catalyst grains, Figure S10: SEM image of the 20%Ni/(18%WO_3_-ZrO_2_) catalyst grains, data on GLC analysis of the liquid products formed during the glycerol hydrogenation over the bifunctional 16%Ni/(20%WO_3_-TiO_2_) catalyst, Figure S11: Chromatogram of the liquid products obtained in the glycerol hydrogenolysis over the 16%Ni/(20%WO_3_-TiO_2_) catalyst in the optimum reaction conditions. Process conditions: Catalyst amount (grains 0.25–0.50 mm)—2 cm^3^/1.6 g; Glycerol concentration in the solution—30% wt.; Glycerol solution supply—1.1 cm^3^/h; Hydrogen feed (NTP)—900 mL/h; Temperature—250 °C; Pressure—31 atm.; Molar H_2_/Glycerol ratio—11.

## Sample Availability

Samples of the catalysts are available from the authors.
