# Supplementary material for: Preparation of Propanols by Glycerol Hydrogenolysis over Bifunctional Nickel-Containing Catalysts
Source: Molecules. 2021 Mar 12;26(6):1565. doi: 10.3390/molecules26061565 (PMC8001030; doi:10.3390/molecules26061565)
Supplement: Supplementary file 1 [file molecules-26-01565-s001.pdf]

Supporting Information

Preparation of Propanols by Glycerol Hydrogenolysis over Biunctional Nickel-Containing Catalysts

Alexander A. Greish [1], Elena D. Finashina [1], Olga P. Tkachenko [1] and Leonid M. Kustov [1,2,3,*]

Supporting Information file includes data for:

DRIFT spectra of CD_3CN desorption from the ZrO_2, TiO_2, 18%WO_3-ZrO_2, 20%WO_3-TiO_2 samples at increased temperature.

In order to evaluate qualitatively the binding energy of CD_3CN with the carrier, IR spectra during a stepwise desorption of CD_3CN from the samples at increase in temperature from 20 to 300°C were obtained (Figures S1-S4). The DRIFT spectra show that of all studied samples, the 20%WO_3-TiO_2 carrier exhibits a most strong adsorption of CD_3CN at a higher temperature. This follows from that when heating this sample in a vacuum at 300°C for 30 minutes, a relatively large quantity of undesorbed D-acetonitrile remains on the carrier surface. According to the binding energy of CD_3CN on the Lewis acid centers at an increased temperature that is favourable for CD_3CN desorption, the samples can be arranged in the following sequence: WO_3-TiO_2 > WO_3-ZrO_2 ≈ TiO_2 > ZrO_2.

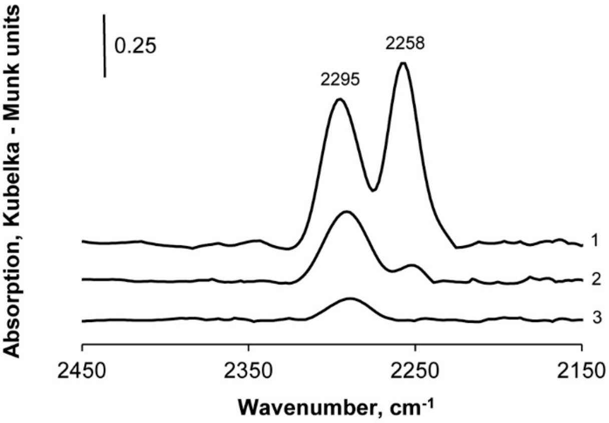

Figure 1. DRIFT spectra of CD_3CN adsorption over ZrO_2. 1 – CD_3CN, 20 °C, 96 mm Hg;. 2 – vacuum, 20°C, 1 h; 3 - vacuum, 100 °C, 30 min.

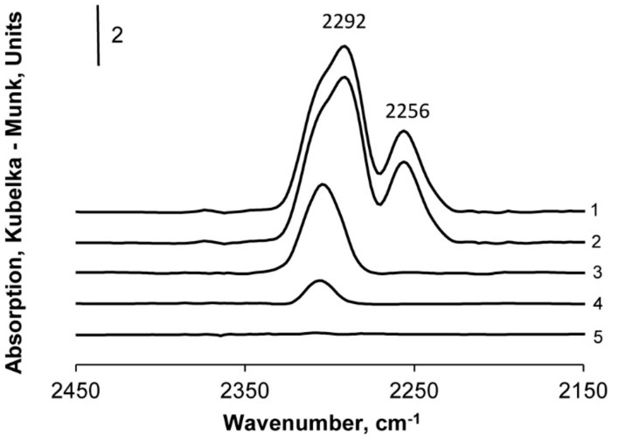

Figure 2. DRIFT spectra of CD_3CN adsorption over 18%WO_3-ZrO_2. 1 – CD_3CN, 20° C, 96 mm Hg; 2 – vacuum, 20° C, 1 h; 3 - vacuum, 100° C, 30 min; 4 - vacuum, 200° C, 30 min; 5 - vacuum, 300° C, 30 min.

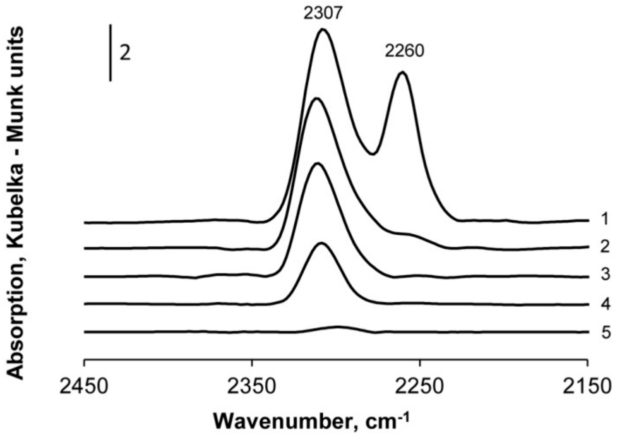

Figure 3. DRIFT spectra of CD_3CN adsorption over TiO_2. 1 – CD_3CN, 20° C, 96 mm Hg;.2 – vacuum, 20° C, 1 h; 3 - vacuum, 100° C, 30 min; 4 - vacuum, 200° C, 30 min; 5 - vacuum, 300° C, 30 min.

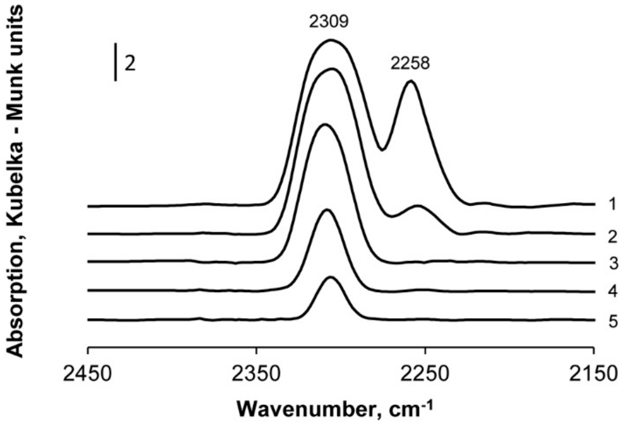

Figure 4. DRIFT spectra of CD_3CN adsorption over 20%WO_3-TiO_2. 1 – CD_3CN, 20° C, 96 mm Hg; 2 – vacuum, 20° C, 1 h; 3 - vacuum, 100° C, 30 min; 4 - vacuum, 200° C, 30 min; 5 - vacuum, 300° C, 30 min.

- DRIFT spectra of the ZrO_2, TiO_2, 18%WO_3-ZrO_2, 20%WO_3-TiO_2 samples in the field of stretching vibrations of OH-groups (4000-3200 cm^{-1}) obtained before and after CD_3CN adsorption.

Additional information about the acidic properties of the studied samples, in particular, the presence of Brønsted acid sites, can be obtained from the analysis of changes in the IR spectra in the field of stretching vibrations of OH-groups (4000-3200 cm^{-1}) associated with CD_3CN adsorption (Figures S4-S8). It is sufficient to compare the IR spectrum of the sample obtained during adsorption of CD_3CN saturated vapor with the IR spectrum of the same sample heated in a vacuum (300°C, 2 h). Comparison of these spectra shows that the adsorption of acetonitrile on almost all samples is accompanied by the formation of hydrogen bonds of acetonitrile with OH groups located on the surface of the oxide carrier, which is a direct proof of the presence of Brønsted acid centers. The strength of Brønsted acid sites can be estimated by the low-frequency shift of the frequency of the OH groups of the sample after adsorption of D-acetonitrile (a wide absorption band with a maximum in the range of 3450-3200 cm^{-1}).

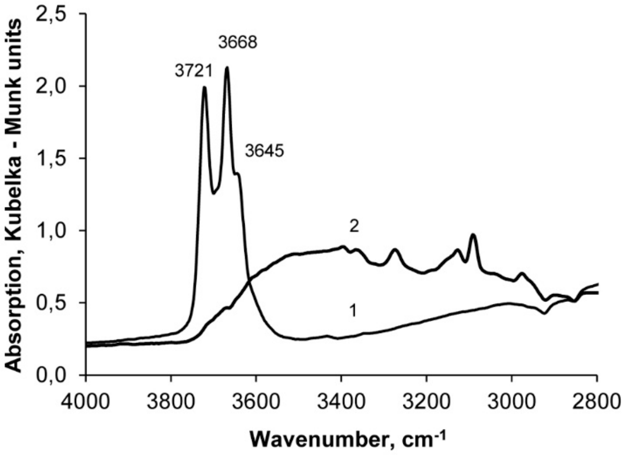

Figure 5. DRIFT spectra of the TiO_2 sample obtained before and after adsorption of CD_3CN. 1 - vacuum, 300° C, 2 h;2 – CD_3CN, 20° C, 96 mm Hg.

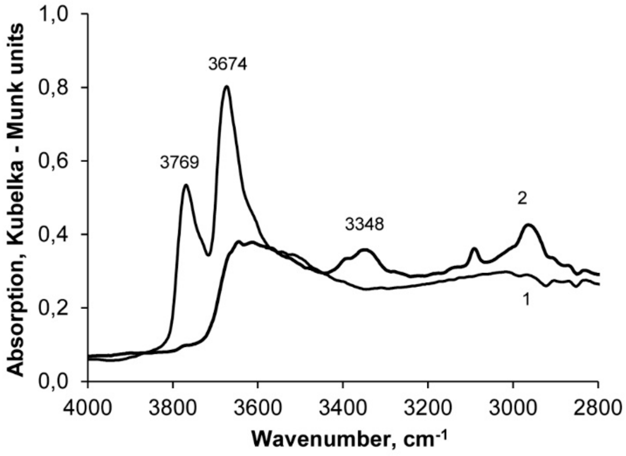

Figure 6. DRIFT spectra of the ZrO_2 sample obtained before and after adsorption of CD_3CN.1 - vacuum, 300° C, 2 h; 2 – CD_3CN, 20° C, 96 mm Hg.

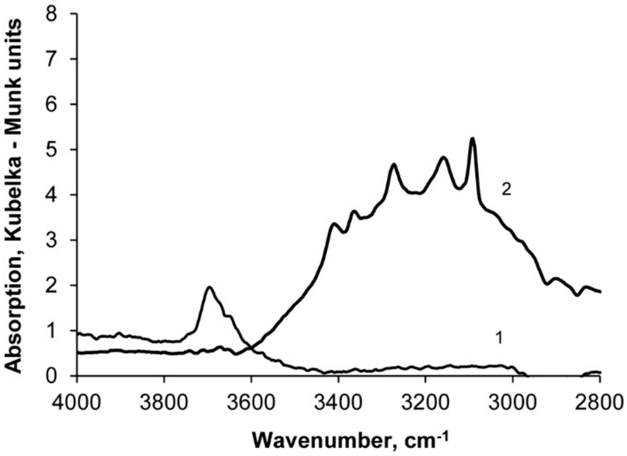

Figure 7. DRIFT spectra of the 20%WO_3-TiO_2 sample obtained before and after adsorption of CD_3CN. 1 - vacuum, 300° C, 2 h; 2 – CD_3CN, 20° C, 96 mm Hg.

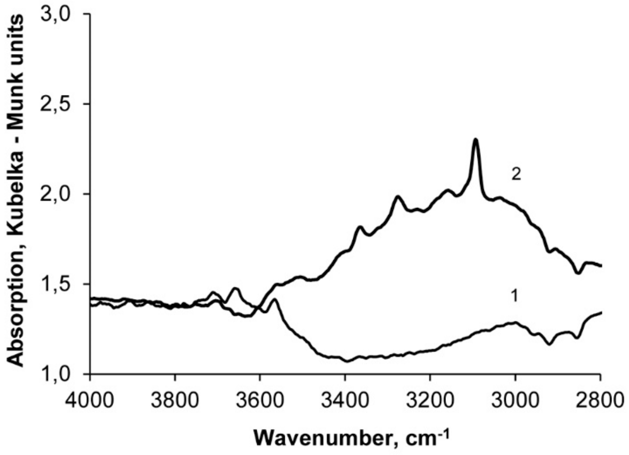

Figure 8. DRIFT spectra of the 18%WO_3-ZrO_2 sample obtained before and after adsorption of CD_3CN. 1 - vacuum, 300° C, 2 h; 2 – CD_3CN, 20° C, 96 mm Hg.

SEM images of the Ni/WO_3-TiO_2 and Ni/WO_3-ZrO_2 catalysts obtained by the scanning electron microscope SNE-3200M (SEC).

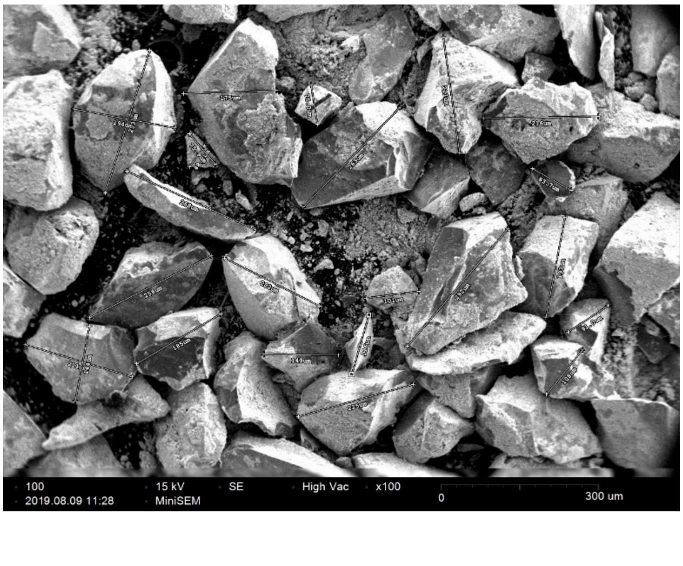

Figure 9. SEM image of the 16%Ni/(20%WO_3-TiO_2) catalyst grains. .

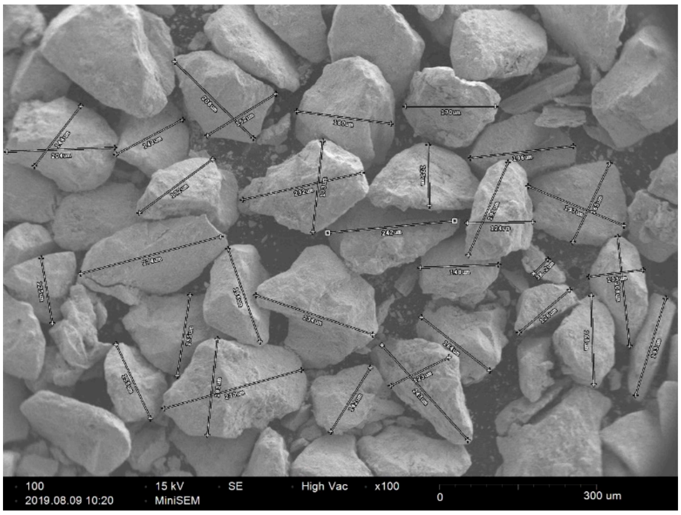

Figure 10. SEM image of the 20%Ni/(18%WO_3-ZrO_2) catalyst grains.

GLC analysis of the liquid products formed during the glycerol hydrogenation over the bifunctional 16%Ni/(20%WO_3-TiO_2) catalyst.

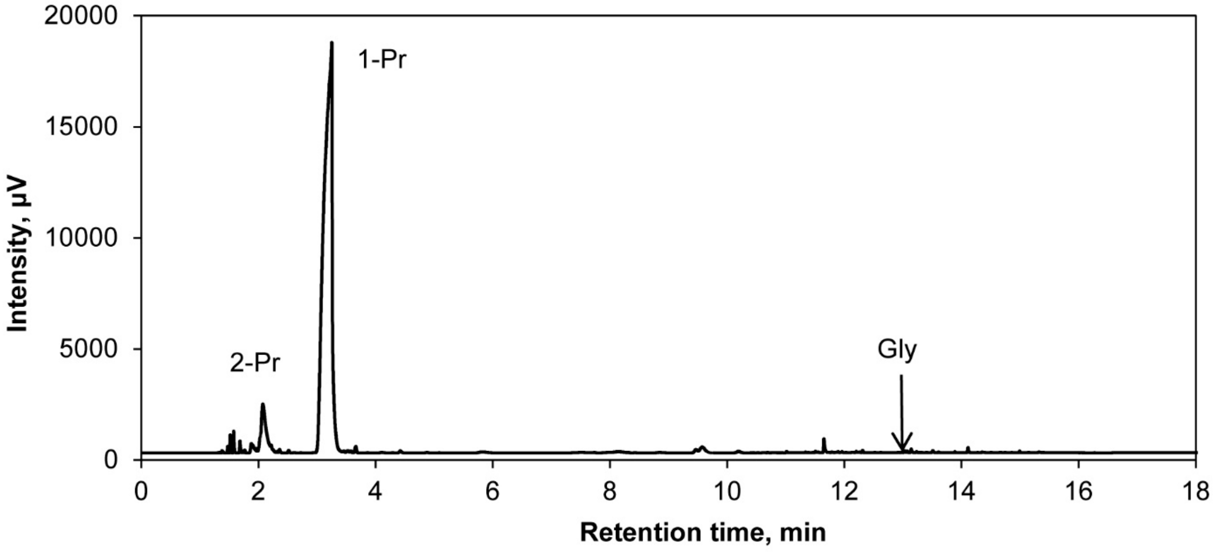

Figure 11. Chromatogram of the liquid products obtained in the glycerol hydrogenolysis over the 16%Ni/(20%WO_3-TiO_2) catalyst in the optimum reaction conditions. Process conditions: Catalyst amount (grains 0.25-0.50 mm) – 2 cm^3/1.6 g; Glycerol concentration in the solution - 30% wt.; Glycerol solution supply – 1.1 cm^3/h; Hydrogen feed (NTP) – 900 ml/h; Temperature – 250°C; Pressure – 31 atm; Molar H_2/Glycerol ratio – 11. .
